# Prevalence of malnutrition and associated factors among older adults from urban and rural residences of Metu district, Southwest Ethiopia

**DOI:** 10.1186/s40795-022-00532-9

**Published:** 2022-05-30

**Authors:** Yohannes Mulu Ferede, Terefe Derso, Mekonnen Sisay

**Affiliations:** 1 Department of Medical Nursing, School of Nursing, College of Medicine and Health Sciences, University of Gondar, Gondar, Ethiopia; 2 Department of Human Nutrition, Institute of Public Health, College of Medicine and Health Sciences, University of Gondar, Gondar, Ethiopia

**Keywords:** Older adults, MNA, Malnutrition, Metu District

## Abstract

**Background:**

In Ethiopia, the proportion of older adults is steadily increasing. This rapidly growing older adult population may increase the burden of malnutrition. However, malnutrition among older adults, particularly those living in rural areas, is regularly underrecognized and/or ignored. There have been no studies among older adults in the Metu district that have used the Min nutritional assessment (MNA). As a result, the purpose of this study is to determine the prevalence of malnutrition and its associated factors among older adults in Metu district, Southwest Ethiopia.

**Methods:**

A community-based comparative cross-sectional study was conducted from May to June 2021 in Metu district. A multi-stage stratified sampling technique was employed. The nutritional status of the older adults was measured by MNA. Data from 616 older adults (308 from urban and 308 from rural residences) was collected through face-to-face interviews using a structured questionnaire. Bivariable and multivariable logistic regression analyses were done to identify factors associated with malnutrition.

**Results:**

According to this study, the overall prevalence of malnutrition in Metu district was 17.5% (95% CI: 14.4%–20.7%). The prevalence of malnutrition in urban and rural residences was 9.9% and 25.2%, respectively. In the overall study, insomnia (AOR: 2.0, 95%CI: 1.1–3.7), residence (AOR: 3.47, 95%CI: 1.8–6.5), and smoking (AOR: 3.7, 95%CI: 2.1–6.7) were associated with malnutrition. In urban residences, depression (AOR: 3.4, 95%CI: 1.2–9.5), dietary diversity score (DDS) (AOR: 3.5, 95%CI: 1.2–10.0), and eating problems (AOR: 2.8, 95%CI: 1.1–7.3) were associated with malnutrition. In rural residence, age (AOR:3.8; 95%CI: 1.2–11), sex (AOR:2.2,95%CI:1.0–4.8), DDS (AOR:5.4,95%CI:2.2–13.3), depression (AOR: 4.6,95%CI:2.2–9.2), and chronic disease (AOR: 3.8 95%CI: 1.8–8.2) were associated with malnutrition.

**Conclusions and recommendations:**

Malnutrition was more prevalent among older adults living in rural areas than in urban areas. In the overall study, insomnia, residence, and smoking were significantly associated with malnutrition. DDS, depression, and eating difficulties were significantly associated with malnutrition among older adults living in urban areas, whereas sex, age, depression, chronic disease, and dietary diversity were factors influencing malnutrition in rural areas. Strengthening strategies aimed at addressing nutrition policy, as well as paying attention to the nutritional needs of the older adult population, can help to improve the health and quality of life of older adults.

## Background

Malnutrition is a condition where energy, protein, and other nutrients are insufficient, excessive, or imbalanced to sustain health, promote cell and tissue growth, and normal organ function, causing detrimental effects on body shape, function, and clinical outcomes [1]. Globally, the prevalence of malnutrition among older adults ranges from 23 to 46% [2]. According to the health and nutrition survey (HANES) data, 16% of community-dwelling Americans over the age of 65 consume fewer than 1000 cal per day, putting them at a high risk of malnutrition [3, 4]. In sub-Saharan Africa, the prevalence of malnutrition among older adults ranges from 6 to 48% [5, 6]. In Ethiopia, malnutrition is a common health problem, affecting 21.9% of the population [7].

Poor nutritional status was prevalent among older adults in both rural and urban settings [8]. In older adults, malnutrition is caused by one or more of the following factors; inadequate food intake, food choices, increased nutrient loss, poor nutrient absorption, loss of appetite, lack of ability to chew and swallow, and increased use of prescription medications [9–13]. Even though diseases, inactivity, and normal aging can cause a gradual loss of muscle mass and strength, leading to malnutrition [14, 15]. This gradual loss of muscle mass and strength below the normal threshold results in functional impairment [16, 17]. Consequently, one aspect of the health-related quality of life diminishes as the individual becomes malnourished. Some of the indicators of health-related quality of life are physical health, mental health, and activity limitation [18].

There is a lack of community-based programs and activities that screen for malnutrition and address those risk factors among vulnerable populations[9]. In Africa, older adults are not considered a priority for targeting nutrition interventions, so the efficacy of different types of nutrition interventions has not been described in this population. Hence, only a handful of nutritionists work with older people, and there are even fewer practitioners who perform high-quality research in their homes. Indeed, nothing else has been attempted, aside from the disaster relief and supplementary feeding programs [19]. Correspondingly, in Ethiopia, malnutrition among older adults is not well detected, and public health research disregards the study of malnutrition among older adults [20].

Therefore, it is vital to assess malnutrition among older adults living in urban and rural residences; to improve their nutritional status, advance living conditions, and develop strategies that are important to address older adult nutritional problems. So, this study aims to assess the prevalence of malnutrition and associated factors among older adults from urban and rural residences in Metu district, Southwest Ethiopia.

## Methods and materials

### Study design and period

A community-based comparative cross-sectional study design was conducted from May to June 2021 in Metu district, Illu Aba Bor zone, Southwest Ethiopia.

### Study setting

Metu district is one of the districts in the Illu Aba Bor Zone, in southwest Ethiopia. It is located 606 km from Addis Ababa, the capital city of Ethiopia. Based on the latest census, it has a population of 108,764 people, of whom 54,768 are men and 53,996 are women; of these, 18,974 are estimated to be older adults. The district has 35 kebeles, including 6 urban kebeles and 29 rural kebeles. It has an elevation of 1605 m and is located at 8°18′N 35°35′E.The area of the district is 687,230 hectares of land. The agro-climates of the residences have a winter dry season and summer rainfall. The main source of income for the indigenous community is agriculture. It is also known for its honey and coffee production. There is one public hospital (Metu Karl referral hospital) in Metu district [21].

### Source and study population

All older adult people aged greater than or equal to 60 years old who lived in Metu district were the source population, whereas older adults randomly selected and living in selected kebeles of Metu district were the study population.

### Inclusion and exclusion criteria

All people aged ≥ 60 years who had lived in the selected kebeles for the past 6 months were included in the study, whereas older adults with visible body edema, unable to respond due to critical illness, or having a mental illness were excluded from the study.

### Sample size and sampling technique

#### Sampling size determination

The Required sample size was first determined by using a double population proportion Formula

$$\frac{{\left(\mathrm{Z\alpha }/2+ {\mathrm{Z}}_{\mathrm{B}}\right)}^{2}[\left({\mathrm{p}}_{1}{\mathrm{q}}_{1}+{\mathrm{p}}_{2}{\mathrm{q}}_{2}\right)}{{\left({\mathrm{p}}_{1}-{\mathrm{p}}_{2}\right)}^{2}}$$

*r* = the sample size n2 to n1 ratio, which is assumed to be 1.

*Z*α/2 = 1.96(type I error).

P1 = the proportion of urban people aged 60 and older who are malnourished.

P2 = the proportion of rural people aged 60 and older who are malnourished.

Using P1 = (17.1% [22] and P2 = 26.6% [20] and adding a 10% non-response rate and multiplying the design effect by 2, a total of 616 older adults (n1 = 308 from urban and n2 = 308 from rural) were enrolled in the study.

The second sample size was calculated by using Epi Info version 4.7. The variables were considered under the following assumptions: 95% confidence level, the ratio of unexposed to exposed (*r* = 1), and 80% power, among those calculated samples; the largest calculated sample size was found to be 600. As we compared the first and second calculated samples, the first method was relatively large, so we took the larger sample size of 616.

#### Sampling technique and procedure

A multi-stage stratified sampling technique was used. The district was stratified by residences into rural and urban kebeles. The residential stratification yields six urban kebeles (Metu town) and 29 rural kebeles (Metu district); among them, two urban and nine rural kebeles, or around 30% of the total kebeles, were randomly selected by using lottery methods. An average of 900 and 1,350 households were found in each urban and rural Kebles, respectively. Likewise, each urban and rural kebele had between 221 and 865 older people. As a result, one household was chosen for every three households in both residences using a systematic random sampling technique. Similarly, participants in the study were chosen using a systematic random sampling method that divided the total number of older adults by the total sample size (538/308) for urban kebeles and (1966/308) for rural kebeles, resulting in participants being chosen every two and six samples, respectively. If participants were not found in the selected households, the samples were taken from the next household. In each household, one participant was selected. If more than one person was found in the selected household, the lotory method was used to select one of the participants.

### Operational definitions

#### Older people

People over the age of sixty are considered older adults [23].

#### Malnourished

Based on the Mini-nutritional assessment (MNA) tool, individuals who had ≥ 17 scores were considered not malnourished and coded as "NO", while those who had a score of less than 17 were considered malnourished and coded as "YES" (which includes normal and at risk of malnutrition) [24].

#### Eating problem

Changes in taste, smell, and appetite generally decline with age, making it more difficult to enjoy eating and keep regular eating habits [25].

#### Geriatric depression scores

(GDS) was used to assess the psychological condition of older adults. A fifteen-item depression scale assessment was used by directly interviewing the respondent. GDS was calculated using 15 yes-or-no items, with yes being for the presence of one of the depression symptoms. Thus, depression was defined using a cut-off point greater than or equal to five [26].

#### Wealth STATUS

Based on the possession of household assets (i.e., refrigerator, sofa, bicycle, television, radio, and mobile telephone), values were computed for each urban and rural area. Finally, the wealth index was ranked into three: poor, medium, and rich by using principal component analysis [20].

#### Dietary diversity score

classification were determined using the Food and Nutrition Technical Assistance (FANTA) description as Low dietary diversity score (DDS): when an older adult person consume < 3 food items per day; Moderate dietary diversity score (DDS): when an older adult consume 4–5 food items per day; High dietary diversity score (DDS): when an older adult consume > 5 food items per day [7].

### Data collection tools

The questionnaire was prepared in English and then translated into Afan Oromo and Amharic. A variety of structured questionnaires were used to collect data, including socio-demographic information, the full mini nutritional assessment (MNA-full), the geriatrics depression scale (GDS), the dietary diversity score (DDS), and others. Malnutrition of older adults was measured by using the Min Nutritional Assessment (MNA) tool [24]. The Mini nutritional assessment tool consists of 18 items with a maximum point value of 30, divided into four categories: general assessment (lifestyle, medication, stress, mobility, neuropsychological problems, and skin lesions); subjective assessment (perceived health and nutritional status); dietary assessment (number of meals, food and fluid intake, and mode of feeding); and anthropometric assessment (weight, height, arm, and calf circumference). Based on this tool, the nutritional status of the participants was classified as either malnourished or not-malnourished. If participants who scored < 17 points were malnourished, and if participants who scored ≥ 17 points were not malnourished (which includes normal and at risk of malnutrition) [24].

The weight of the study participants was measured using a beam balance to the nearest 0.1 kg without shoes and all heavy clothing, including jackets, jerseys, and belts. The weighing scale was checked against a standard weight each day for its accuracy. Calibration was performed before weighing each study participant by setting it to zero.

The height of the study participants was taken using an adult stadiometer for those who could stand. For those who are unable to stand, the arm span from the sterna notch to the tip of the finger or knee height was to be used as a proxy indicator for the height of the subjects using a specific formula for the specific sex. Participants were asked to take off their shoes and stand on the Frankfurt plane (stand erect and look straight ahead on the horizontal plane). The occipital (back of the head), shoulder blades, buttocks, and heels were touched by the measuring board, and height was recorded to the nearest 0.01 cm.

The BMI was calculated by dividing the weight in kg by the height in meters squared and was expressed in kg/m2. However, when the height measurement was not possible, calf circumference was used instead of BMI.

The Geriatric Depression Scale item 15 (GDS-15) was used to assess depression among older adults as suggested by the Royal College of Physicians, the British Geriatric Society, and the Royal College of General Practitioners. Thus, depression was defined using a cut-off point greater than or equal to five.

The short twenty-four-hour dietary recall was used to assess the dietary intake pattern of the clients, as it reduces the recall bias secondary to a memory lapse. Cronbach's Alpha was done to check the internal consistency of the tools, and each item scored an acceptable level of reliability. The data was collected daily. Data collectors were approached by introducing themselves to and interviewing the selected respondents after informed consent was obtained.

### Data quality assurance

An interviewer-administered structured questionnaire and anthropometric measurements were used to collect data from interviewees. Through house to house visits, an interviewer-administered structured questionnaire was used to collect socio-demographic, health and lifestyle, geriatrics depression scale (GDS), dietary diversity score (DDS), and nutrition or dietary habits characteristics of older adults.

Eight diploma nurses who can sufficiently speak and understand both Afan Oromo and Amharic were recruited as interviewers, and three BSc nurses were recruited as supervisors. For one day, supervisors were trained on the details of the questionnaire, how to control the quality of data, and the efficiency of data collectors.

Data collectors were trained for one day in appropriate interview techniques and anthropometric measurements like height and weight. Practices were performed before the actual data collection. After that, constructive feedback was given to the data collectors by the investigators and supervisor until they became clear of the data collection procedures and instruments.

A consent form was provided for the study subject to those who voluntarily agreed to participate. Data collectors have interviewed study subjects in their homes. When study subjects were not present during data collection, the interviewer revisited the house at different time intervals, at least three times since it is a community-based study. Strict daily supervision of the data collection process was maintained throughout the data collection period. The time estimated to complete the interview was 25 to 35 min. Short and precise information regarding malnutrition prevention was provided to all participants at the end of the interview.

### Data analysis and processing

Data were entered into Epi-info statistical software version 7 before being exported to the Statistical Package for Social Science (SPSS) version 22 for analysis, with three separate logistic regression models (urban, rural, and both urban and rural combined) fitted. Frequencies and cross-tabulations were used to summarize descriptive statistics of the data, and tables were used for data presentation.

The significance of the association was determined using crude and adjusted odds ratios with 95% confidence intervals. The Hosmer and Lemeshow goodness of fit of overall malnutrition in urban and rural residences was 0.649, as well as 0.204 in urban and 0.287 in rural residences. All variables that were less than 0.2 during bivariable analysis were considered as candidates for multiple logistic regressions. After adjusting for their effect on the outcome variable, those variables with a *p*-value < 0.05 with a 95% confidence interval were regarded as factors significantly associated with malnutrition.

## Results

### Socio-demographic and economic characteristics of respondents in Metu district

A total of 610 older adults participated in this study, with a 99.0% response rate. Of the total participants, 304 and 306 were from urban and rural residences, respectively. About 293 (48.0%) of the study participants were male. Of these, 160 (22.4%) were from rural residences and 133 (43.5%) of them were from rural residences. Concerning marital status, 187 (61.5%) and 195 (63.7%) of older adults were married in urban and rural residences respectively. Regarding educational status, 174 (57.2%) urban older adults had a higher formal education than rural older adults 5 (1.6%). Regarding their main source of financial support, 91 (31.9%) of older adults living in urban residences were on pensions, while 181 (59.2%) of rural older adults were from agriculture (Table 1).

**Table 1:** Socio-demographic characteristics of prevalence and associated factors among older adults in urban and rural residences of Metu district, southwestern, Ethiopia, 2021 (*n* = 610)

Characteristics	Category	Residence		
		Urban	Rural	Overall
		N (%)	N (%)	N (%)
Age	60–64	114(37.5%)	110(35.9%)	224(36.7%)
	65 – 74	122(40.1%)	158(51.6%)	280(45.9%)
	** > ** = 75	68(22.4%)	38(12.4%)	106(17.4%)
Sex	Male	160(22.4%)	133(43.5%)	293(48.0%)
	Female	144(47.4%)	173(56.5%)	317(52.0%)
Residence	Residence	304(49.2%)	306(52.8%)	610(100.0%)
Marital status	Married	187(61.5%)	195(63.7%)	382(62.6%)
	Others^a^	117(38.5%)	111(36.3%)	228(37.4%)
Head of Household	Father	128(42.1%)	117(38.2%)	245(40.2%)
	Mother	97(31.9%)	84(27.5%)	181(29.7%)
	Children/relative	79(26.0%)	105(34.3%)	184(30.2%)
Religion	Orthodox	163(53.6%)	156(51.0%)	319(52.3%)
	Muslim	88(28.9%)	110(35.9%)	198(32.5%)
	Protestant	53(17.4%)	40(13.1%)	93(15.2%)
Educational status	No formal education	27(8.9%)	152(49.7%)	179(29.3%)
	Primary	26(8.6%)	113(36.9%)	139(22.8%)
	Secondary	77(25.3%)	36(11.8%)	113(18.5%)
	Certificate and diploma	174(57.2%)	5(1.6%)	179(29.3%)
Occupation	House wife	35(11.5%)	133(43.5%)	168(27.5%)
	Pension	105(34.5%)	2(7.0%)	107(17.5%)
	Private organization	78(25.7%)	0(0%)	78(12.8%)
	Private work	86(28.3%)	1(3%)	87(14.3%)
	Farmer	0	170(27.9%)	170(27.9%)
Family size	1 – 3	140(46.1%)	102(33.3%)	242(39.7%)
	4 – 6	164(53.9%)	204(66.7%)	368(60.3%)
The main source of financial support	pension	97(31.9%)	17(5.6%)	114(18.7%)
	Family support	91(29.9%)	108(35.3%)	199(32.9%)
	Others^b^	116(38.2%)	181(59.2%)	297(48.7%)
Wealth index	Poor	121(39.8%)	6(2.0%)	127(20.8%)
	Medium	61(20.1%)	232(75.8%)	293(48.0%)
	Rich	122(40.1%)	68(22.2%)	190(31.1%)

^a^Single/Divorced/Widowed

^b^Organization support/Agriculture

### Health and lifestyle characteristics of respondents in Metu district

About, 42.8% of rural older adults and 30.6% of urban older adults reported having at least one chronic disease, with 108 (35.3%) and 89 (29.3%) having a history of hospitalization in the previous year, respectively. Regarding lifestyle characteristics, 22.0% of urban and 29.7% of rural older adults had the habit of smoking cigarettes. However, only 28.6% of urban older adults reported eating problems, whereas 32% of rural older adults reported eating problems (Table 2).

**Table 2:** Health and lifestyle characteristics of older adults in Metu district, Illu Aba Bor zone, Southwestern Ethiopia, 2021 (*n* = 610)

Characteristics	Category	Residence		
		Urban	Rural	Overall
		N (%)	N (%)	N (%)
Chronic disease	Yes	93(30.6%)	131(42.8%)	224(36.7%)
	No	211(69.4%)	175(57.2%)	386(63.3%)
Hospitalization last year	Yes	89(29.3%)	108(35.3%)	197(32.3%)
	No	215(70.7%)	198(64.7%)	413(67.7%)
Insomnia	Yes	31(10.2%)	132(43.1%)	163(26.7%)
	No	273(89.8%)	174(56.9%)	447(73.3%)
Eating problem	Yes	87(28.6%)	98(32.0%)	185(30.3%)
	No	217(71.4%)	208(68.0%)	425(69.7%)
Edentulous	Yes	140(46.1%)	196(64.1%)	336(55.1%)
	No	164(53.9%)	110(35.9%)	274(44.9%)
Denature	Yes	43(14.1%)	13(4.2%)	56(9.2%)
	No	261(85.9%)	293(95.8%)	554(90.8%)
Smoking	Yes	67(22.0%)	91(29.7%)	158(25.9%)
	No	237(78.0%)	215(70.3%)	452(74.1%)

### Dietary Diversity Score (DDS) of older adults in Metu district

The most commonly consumed food groups by urban older adults in the last 24 h were cereals and grain food groups 222 (73.0%), followed by spices, condiments, and beverages 212 (69.7%). While for rural residences, dark green leafy vegetables (191 (62.4%), followed by spices and condiments, 185 (60.5%) of the food groups were consumed. Regarding the dietary diversity score (DDS), 18.4%, 18.8%, and 62.8% scored low, moderate, and high for urban respectively. In rural, 25.2%, 28.5%, and 46.4% scored low, moderate, and high, respectively (Table 3).

**Table 3:** The food groups eaten by older adults during the past day (24 h) of the survey in Metu district, Southwestern Ethiopia, 2021 (*n* = 610)

Food Groups	Residence		
	Urban N (%)	Rural N (%)	Overall N (%)
Cereals and grains	222(73.0%)	174(56.9%)	369(64.9%)
White roots and tubers	134(44.1%)	47(15.4%)	181(29.7%)
Legumes, nuts, and seeds	146(48.0%)	153(50.0%)	299(49.0%)
Oils and fats	159(52.3%)	46(15.0%)	205(33.6%)
Milk and milk products	155(51.0%)	112(36.6%)	267(43.8%)
Flesh Meats	39(12.8%)	28(9.2%)	67(11.0%)
Eggs	151(49.7%)	76(24.8%)	227(37.2%)
Dark green leafy vegetables	207(68.1%)	191(62.4%)	398(65.2%)
Vitamin A-rich fruits	135(44.4%)	64(20.9%)	199(32.6%)
Other vegetables	174(57.2%)	167(54.6%)	341(55.9%)
Other fruits	97(31.9%)	56(18.3%)	153(25.1%)
Organ meats	62(20.4%)	44(14.4%)	106(17.4%)
Vitamin A-rich vegetables and tubers	163(53.6%)	145(47.4%)	308(50.5%)
Sweets	208(68.4%)	155(50.7%)	363(59.5%)
Spices Condiments beverages	212(69.7%)	185(60.5%)	397(65.1%)
Fish and seafood	6(2.0%)	34(11.1%)	40(6.6%)
Dietary diversity score			
Low	56(18.4%)	77(25.2%)	133(21.8%)
Moderate	57(18.8%)	87(28.5%)	144(23.6%)
High	191(62.8%)	142(46.4%)	333(54.6%)

### Depression status of an older adult by residence in Metu district

Among the study participants 98 (32.2%) of older adults living in an urban residence, and 103 (33.7%) of older adults living in rural residences had depression (Table 4).

**Table 4:** A detailed report on the outcomes of GDS short by residence form screening for older adult people in Metu district, Southwestern Ethiopia, 2021 (*n* = 610)

GDS dimensions	Category	Residence		
		Urban N (%)	Rural N (%)	Overall N (%)
GDS	Not depressed	206(67.8%)	203(66.3%)	409(67.0)
	depressed	98(32.2%)	103(33.7%)	201(33.0%)
Are You satisfied with your life?	Yes	247(81.3%)	214(69.9%)	461(75.6%)
	No	57(18.8%)	92(30.1%)	149(24.4%)
Have you dropped many of your activities and interests?	Yes	93(30.6%)	96(31.6%)	189(31.0%)
	No	211(69.4%)	210(68.6%)	421(69.0%)
Do you feel that your life is empty?	Yes	71(23.4%)	98(32.0%)	169(27.7%)
	No	233(76.6%)	208(68.0%)	441(72.3%)
Do you often have bored?	Yes	83(27.3%)	110(35.9%)	193(31.6%)
	No	221(72.2%)	196(64.1%)	417(68.4%)
Are you in a good spirit most of the time?	Yes	250(82.2%)	206(67.3%)	456(74.8%)
	No	54(17.8%)	100(32.7%)	154(25.2%)
Are you afraid that something bad is going to happen to you?	Yes	62(20.4%)	101(33.0%)	163(26.7%)
	No	242(79.6%)	205(67.0%)	447(73.3%)
Do you feel happy most of the time	Yes	226(74.3%)	208(68.0%)	434(71.1%)
	No	78(25.7%)	98(32.0%)	176(28.9%)
Do you often feel helpless?	Yes	78(25.7%)	96(31.4%)	174(28.5%)
	No	226(74.3%)	210(68.6%)	436(71.5%)
Do you prefer to stay at home rather than gogo out?	Yes	167(54.9%)	216(70.6%)	383(62.8%)
	No	137(45.1%)	90(29.4%)	227(37.2%)
Do you feel that you have more problemsproblems with memory than most?	Yes	172(56.6%)	182(59.5%)	354(58.0%)
	No	132(43.4%)	124(40.5%)	256(42.0%)
Do you think it is wonderful to be alive now?	Yes	255(83.9%)	233(76.1%)	488(80.0%)
	No	49(16.1%)	73(23.7%)	122(20.0%)
Do you feel pretty worthless the way you are now?	Yes	60(19.7%)	55(18.0%)	115(18.9%)
	No	244(80.3%)	251(82.0%)	495(81.1%)
Do you feel full of energy?	Yes	251(82.6%)	218(71.2%)	469(76.9%)
	No	53(17.4%)	88(28.8%)	141(23.1%)
Do you feel that your situation is hopeless?	Yes	71(23.4%)	74(24.2%)	145(23.8%)
	No	233(76.6%)	232(75.8%)	465(76.2%)
Do you think that most people are better off than you are?	Yes	75(24.7%)	93(30.4%)	168(27.5%)
	No	229(75.3%)	213(69.6%)	442(72.5%)

### Prevalence of malnutrition among older adults in Metu district

According to this study, the overall prevalence of malnutrition in Metu district was 17.5% (95% CI: 14.4%—20.7%). The prevalence of malnutrition in urban and rural residences was 9.9% (95% CI: 6.3—13.7%) and 25.2% (95% CI: 20.4—30.4%) respectively. More than half of the study participants had eaten three full meals a day 271 (89.1%) and 168 (54.9%) in both urban and rural residences respectively. And had no decrease in food intake 257 (84.5%) and 185 (60.5%) for the past 3 months in both urban and rural residences respectively.

On the other hand, the majority (98.4%) and (97.1%) of older adults in urban and rural residences did not consume selected consumption markers of protein like dairy products (milk, cheese, and yogurt), legumes, eggs, and poultry every day respectively. 58 (19.1%) of Urban older adults and 75 (24.5%) of rural older adults fed themselves without any difficulty (Table 5).

**Table 5:** Nutritional status of older adults in Metu district, Southwestern Ethiopia, 2021 (*n* = 610)

Variables	Residence		
	Urban N (%)	Rural N (%)	Overall N (%)
Malnutrition			
Yes	30(9.9%)	77(25.2%)	107(17.5%)
No	274(90.1%)	229(74.8%)	503(82.5%)
Has food intake declined over the past 3 months			
Yes	47(15.5%)	121(39.5%)	168(27.5%)
No	257(84.5%)	185(60.5%)	442(72.5%)
Weight loss during the last 3 months			
Weight loss greater than 3 kg	4(1.3%)	6(2.0%)	10(1.6%)
Dose not know	41(13.5%)	178(58.2%)	219(35.9%)
Weight loss between 1 and 3 kg	0	7(2.3%)	7(1.1%)
No Weight loss	259(85.2%)	115(37.6%)	374(61.3%)
Mobility			
Bed or chair bound	8(2.6%)	4(1.3%)	12(2.0%)
Able to get out of bed /chair but does not go out	27(8.9%)	47(15.4%)	74(12.1%)
goes out	269(88.5%)	255(83.3%)	524(85.9%)
Has suffered psychological stress or acute disease in the past 3 months			
Yes	35(11.5%)	53(17.3%)	88(14.4%)
No	269(88.5%)	253(82.7%)	522(85.6%)
Neuropsychological problem			
Severe dementia or depression	19(6.3%)	4(1.3%)	23(3.8%)
Mild dementia	12(3.9%)	44(14.4%)	56(9.2%)
No psychological problem	273(89.8%)	258(84.3%)	531(87.0%)
Body Mass Index (BMI)			
BMI less than 19	32(10.5%)	59(19.3%)	91(14.9%)
BMI 19 to less than 21	75(24.7%)	70(22.9%)	145(23.8%)
BMI 21 to less than 23	126(41.4%)	118(38.6%)	244(40.0%)
BMI 23 or greater	71(23.4%)	59(19.3%)	130(21.3%)
Lives independently (not in a nursing home or hospital			
Yes	304(100.0%)	306(100.0%)	610(100.0%)
No	0	0	0(0.0%)
Takes more than 3 prescription drugs per day			
Yes	22(7.2%)	19(6.2%)	41(6.7%)
No	282(92.8%)	287(93.8%)	569(93.3%)
Pressure sores or skin ulcers			
Yes	0	0	0
No	304(100.0%)	306(100.0%)	610(100.0%)
How many full meals does the client eat daily			
1 Meal	6(2.0%)	16(5.2%)	22(3.6%)
2 Meal	27(8.9%)	122(39.9%)	149(24.4%)
3 Meal	271(89.1%)	168(54.9%)	439(72.0%)
Selected consumption markers for protein intake (Meat, fish, or poultry every day)			
No	299(98.4%)	297(97.1%)	596(97.7%)
Yes	5(1.6%)	9(2.9%)	14(2.3%)
Consume two or more servings of fruit or vegetable per day			
Yes	66(10.8%)	44(7.2%)	110(18.1%)
No	238(39.0%)	278(45.5%)	500(81.9%)
How much fluid (water, juice, coffee, tea, milk..) is consumed per day			
Less than 3 cups	41(13.5%)	102(33.3%)	143(23.4%)
3 to 5 cups	44(14.5%)	184(60.1%)	228(37.4%)
More than 5 cups	219(72.0%)	20(6.5%)	239(39.2%)
Mode of feeding			
Self–fed with some difficulty	58(19.1%)	75(24.5%)	133(21.8%)
Self–fed without any problem	246(85.2%)	231(75.5%)	477(78.2%)
Self–view of nutritional status			
View self as being malnourished	34(11.2%)	19(6.2%)	53(8.7%)
Is uncertain of nutritional status	39(12.8%)	160(52.3%)	199(32.6%)
View self as having no nutritional problem	231(76.0%)	127(41.5%)	358(58.7%)
In comparison with other people of the same age, how does the client consider his /her health status?			
Not as good	29(9.5%)	33(10.8%)	62(10.2%)
Dose not know	11(3.6%)	165(53.9%)	176(28.9%)
As good	76(25.0%)	64(20.9%)	140(23.0%)
Better	188(61.8%)	44(14.4%)	232(38.0%)
Mid–arm circumference (MAC) in cc			
MUAC less than 21	48(15.8%)	65(21.2%)	113(18.5%)
MUAC 21 to 22	63(20.7%)	52(17.0%)	115(18.9%)
MUAC greater than 22	193(63.5%)	189(61.8%)	382(62.6%)
Calf circumference (CC) in cm			
CC less than 31	60(20.1%)	97(31.7%)	158(25.9%)
CC 31 or greater	243(79.9%)	209(68.3%)	452(74.1%)

### Factors associated with malnutrition among older adults in urban residences of Metu district

In the bivariable logistic regression analysis; marital status, educational status, depression, dietary diversity, smoking, chronic disease, hospitalization in the previous year, and eating problems were associated with malnutrition.

In the multivariable logistic regression analysis; DDS, eating problems, and depression were significantly associated with malnutrition. The odds of malnutrition were 3.5 times higher among older adults with low dietary diversity than high dietary diversity (AOR = 3.5, 95% CI = 1.2–10.0). Older adults who had an eating problem were 2.8 times more likely malnourished than their counterpart (AOR = 2.8, 95% CI: 1.1–7.3) and the odds of malnutrition was 3.4 times higher among older adults who had depression than older adults who were free of depression (AOR = 3.4, 95% CI: 1.2–9.59) (Table 6).

**Table 6:** Multivariable logistic regression result demonstrating the association of malnutrition (malnourished/Not) with independent variables based on MNA scores among older adults in Metu district, Southwestern Ethiopia, in 2021

Variables	Category	Malnourished		COR (95% CI)	AOR (95%)	*P*-value
		No	Yes			
Marital status	Married	174	13	1	1	
	Others^a^	100	17	2.27(0.09–4.87)	3.07(0.76–7.55)	0.314
DDS	Low	40	16	9.15(3.66–22.84)	3.54(1.24–10.07)*	0.017
	Moderate	51	6	2.69(0.89–8.11)	0.78(0.22–2.74)	
	High	183	8	1	1	
Eating problem	No	207	10	1	1	
	Yes	67	20	6.17(2.75–13.85)	2.84(1.10–7.36)*	0.031
Smoking	No	222	15	1	1	
	Yes	52	15	4.26(1.9–9.2)	1.0(0.4–3.0)	0.857
Hospitalization last year	No	204	11	1	1	
	Yes	70	19	5.03(2.28–11.09)	3.46(1.45–8.32)	0.053
Chronic disease	No	195	16			
	Yes	79	14	2.16(1.0–4.6)	0.96(0.3–2.5)	0.935
Depression	No depression	198	8	1	1	
	depression	76	22	7.16(3.05–16.78)	3.40(1.20–9.59)*	0.021

^a^Single/Divorced/widowed

*COR* Crude odd ratio, *CI* Confidence interval, *AOR* Adjusted odd ratio

1: reference Category;*: significant at *p* < 0.05;**: significant at *p* < 0.001

### Factors associated with malnutrition among older adults in rural residences of Metu district

Variables like age, sex, marital status, educational status, meal, chronic disease, eating problems, previous hospitalization, dietary diversity, and depression were significantly associated with malnutrition in the bivariable logistic regression analysis.

In multivariable logistic regression analysis; sex, age, chronic disease, DDS, and depression were significantly associated with malnutrition among older adults living in a rural residence. Females were 2.2 times more likely to develop malnutrition as compared with male older adults (AOR 2.26; 95% CI: 1.06–4.8). Likewise, the odds of malnutrition were 3.81 times higher among older adults age 75 years old or above as compared with older adults age 65–74 years old (AOR: 3.81- 95% CI: 1.24–11.70). The likelihood of being malnourished was 3.8 times higher among older adults who had the chronic disease (AOR: 3.85, 95% CI: 1.80–8.23). Dietary diversity is one of the factors which affects malnutrition; the odds of malnutrition was 5.44 times higher among older adults who had low dietary diversity as compared with older adults who had high dietary diversity (AOR: 54.4; 95% CI: 2.21–13.39). And the odds of malnutrition were 4.6 times higher among older adults who had depression than older adults who were free of depression (AOR: 4.60, 95% CI: 2.28–9.26) (Table 7).

**Table 7:** Multivariable logistic regression result demonstrating the association of malnutrition (malnourished or not) with independent variables based on MNA scores among older adults living in rural Metu district, southwestern Ethiopia, in 2021

Variables	category	Malnourished		COR (95% CI)	AOR (95%)	*P* value
		No	Yes			
Sex	Male	114	19	1	1	
	Female	115	58	3.02(1.69–5.40)	2.26(1.06–4.82)*	0.034
Age	60—64	92	18	1	1	
	65—74	124	34	1.40(0.74–2.63)	0.86(0.38–1.93)	
	> = 75	13	25	9.82(4.24–22.75)	3.81(1.24–11.70)*	0.019
Chronic disease	No	159	16	1	1	
	Yes	70	61	8.66(4.66–16.06)	3.85(1.80–8.23)**	0.000
Insomnia	No	141	33	1	1	
	Yes	88	44	2.13(1.2–3.6)	1.80(0.8–3.6)	0.104
DDS	Low	38	39	10.18(4.93–21.01)	5.44(2.21–13.39)**	0.000
	Moderate	62	25	4.00(1.91–8.34)	3.50(1.45–8.45)	0.005
	High	129	13	1	1	
Eating problem	No	178	30	1	1	
	Yes	51	47	5.46(3.1–9.5)	1.90(0.7–4.5)	0.152
Marital status	married	167	28	1	1	
	Others^a^	62	49	4.71(2.71–8.15)	2.14(0.52–4.36)	0.336
Depression	No depression	182	21	1	1	
	depression	47	56	10.32(5.69–18.72)	4.60(2.28–9.26)**	0.000

^a^Single, Divorced, widowed

*COR* Crude odds ratio, *CI* Confidence interval, *AOR* Adjusted odds ratio

1: reference Category: *significant at *p* < 0.005;**significant at *p* < 0.001

### Factors associated with the overall malnutrition of older adults in Metu District

In the bivariable logistic regression analysis, factors like; age, sex, marital status, residence, educational status, source of finance, smoking, chronic disease, insomnia, eating problems, previous hospitalization, dietary diversity, depression, were significantly associated with malnutrition.

In the multivariable logistic regression analysis; sex, residence, eating problem, insomnia, cigarette smoking, and depression were statistically associated with malnutrition. The odds of malnutrition were 2.46 times higher among females than male older adults (AOR: 2.46, 95% CI: 1.3–4.4). Respective to residence; the likely hood of malnutrition was 3.4 times higher among older adults who lived in rural residences than urban residences (AOR: 3.4; 95% CI: 1.8–6.5). Eating problem was also the other problem affecting malnutrition; the odds of malnutrition was 2.47 time's higher among older adults who had an eating problem as compared with free of eating problem (AOR: 2.47, CI: 1.3–4.4). Cigarette smokers were 3.77 times more likely malnutrition as compared with non-smokers (AOR = 3.77, 95% CI: 2.1–6.7)**.** The likelihood of malnutrition was 2.07 times higher among older adults who had insomnia as compared with older adults who were free of insomnia (AOR: 2.07; 95% CI: 1.1–3.7). Also, the odds of malnutrition were 3.75 times higher among older adults who had depression than older adults who were free of depression (AOR: 3.75, 95% CI: 2.0–6.7) (Table 8).

**Table 8:** Overall, bivariable and multivariable logistic regression analysis of factors associated with malnutrition among older adults in Metu district, southwestern Ethiopia, 2021

Variables	Category	Malnourished		COR(95% CI)	AOR (95% CI)	*P*-Value
		No	Yes			
Sex	Male	259	34	1	1	
	Female	244	73	2.27(1.4–3.5)	2.46(1.3–4.4)*	0.003
Marital status	Married	341	41	1	1	
	Others^a^	162	66	3.38(2.1–5.2)	3.11(0.87–5.4)	0.602
Residence	Urban	274	30		1	
	Rural	229	77	3.07(1.9–4.8)	3.47(1.8–6.5)^a^	0.000
Chronic disease	No	354	32	1	1	
	Yes	149	75	5.56(3.5–8.7)	1.47(0.7–2.7)	0.219
Eating problem	No	385	40	1	1	
	Yes	118	67	5.46(3.5–8.5)	2.47(1.3–4.4)*	0.002
Hospitalization last year	No	365	48	1	1	
	Yes	138	59	3.25(2.1–4.9)	1.77(0.99–3.1)	0.051
Insomnia	No	394	53	1	1	
	yes	109	54	3.68(2.3–5.6)	2.07(1.1–3.7)*	0.017
Smoking	No	407	45	1	1	
	Yes	96	62	5.84(3.7–9.1)	3.77(2.1–6.7)**	0.000
Depression	No depression	380	29	1	1	
	depression	123	78	8.31(5.1–13.3)	3.75(2.0–6.7)**	0.000
DDS	Low	78	55	10.4(5.9–18.3)	4.3(2.1–8.8)**	0.000
	Moderate	113	31	4.07(2.2–7.3)	1.84(0.9–3.7)	
	High	312	21	1	1	

^a^Single/Divorced/Widowed

*COR* Crude odds ratio, *CI* Confidence interval, *AOR* Adjusted odds ratio

### Discussion

According to this study, the overall prevalence of malnutrition in Metu district was 17.5% (95% CI: 14.4%–20.7%). The prevalence of malnutrition in urban and rural residences was 9.9% (95% CI: 6.3—13.7%) and 25.2% (95% CI: 20.4—30.4%) respectively. The overall finding was in line with the study done in Harar, Ethiopia (15.7%) [27] and the study done in Egypt (17.9%) [6]. Besides, the finding was lower than the study done in Nairobi, Machakos (29.8%), and Uganda (22.5%). The discrepancy could be attributed to a population difference or geographical variation and it might be due to age group differences [28]. On the other hand, the current study was slightly higher than the study conducted in Senegal (16.7%) [29] and Kenya, Dagoretti district (11.4%) [28]. The disparity could be attributed to differences in nutritional assessment methods as well as geographical variation. Furthermore, research findings in Senegal and Kenya used the Body Mass Index (BMI) to assess malnutrition, whereas this study used the Mini Nutritional Assessment tool to assess nutritional problems [30].

The current study compares the prevalence of malnutrition among older adults in Metu district between urban and rural residences. As a result, malnutrition among older adults in urban areas was 9.9%, while malnutrition among older adults in rural areas was 25.2%. A study conducted in Senegal found that 10.5% of older adults living in urban areas were malnourished, while 23.5 percent of older adults living in rural areas were malnourished. [29]. It was supported by the studies done in Benin, where malnutrition was found to be 17.0% and 22.2% in urban and rural older adults, respectively [28]. Furthermore, it was supported by a study conducted in Harar, Ethiopia, which found that older adults in rural residences had a higher proportion of malnutrition 17.1% than older adults in urban residences.4.9% [27].

In terms of associated factors, rural residents were found to be at a higher risk of malnutrition than urban residents. According to the findings of this study, the likelihood of malnutrition was 3.4 times higher among older adults living in rural areas than in urban areas. This finding was consistent with the findings of a study conducted in France. [31]. This might be attributed to the difference in socioeconomic status and dietary habits, these puts rural residences at risk of malnutrition.

Furthermore, the likelihood of malnutrition was 2.07 times higher in older adults who had insomnia compared to older adults who did not have insomnia. This findings agree with studies done in Lebanon [32] and Turkey [33]. This could be due to a lack of sleep, which increases energy consumption and contributes to malnutrition, and/or insomnia, which increases the risk of falls, cognitive impairment, and depression. These conditions, when combined, raise the risk of malnutrition [34].

Besides that, smoking is another factor that has been linked to malnutrition. Thus, cigarette smokers were 3.77 times more likely to suffer from malnutrition than nonsmokers. This finding was supported by a study conducted in Taiwan [35] and Cuba [36]. The possible justification could be that smoking is one of the risk factors for malnutrition. [37].

In rural areas, the likelihood of older adults being malnourished increases with age. As a result, the odds of malnutrition were 3.81 times higher in adults 75 years or older compared to older adults 65–74 years old. This finding was consistent with the data reported in Senegal [28], Addis Ababa, Ethiopia [20], and Gonder, Ethiopia [7]. This could be due to a change in taste and smell, as well as a general decline in appetite with age, making it more difficult to enjoy eating and maintain regular eating habits, likely to result in malnutrition [25].

In rural residences, females were 2.2 times more likely to develop malnutrition as compared with male older adults. This result was supported by the study done in rural India [38] and Gonder, Ethiopia [7]. This could be because women are more responsible for the care of household members and are more financially reliant on family members than men.

Correspondingly, depression was found to be a factor for both people living in urban and rural areas. In urban areas, the odds of malnutrition were 3.4 times higher among depressed older adults than among non-depressed older adults, and in rural areas, the odds of malnutrition were 4.6 times higher among depressed older adults than among non-depressed older adults. This finding was consistent with the results of the Addis Abeba study [20], India [39], and Italy [40]. The possible justification could be that depression is one of the risk factors for malnutrition, which decreases food intake [25].

This research revealed that dietary diversity scores were strongly associated to malnutrition in both urban and rural areas. In urban areas, the odds of malnutrition were 3.5 times higher among older adults with low dietary diversity compared to those with high dietary diversity, and in rural areas, the odds of malnutrition were 5.44 times higher among older adults with low dietary diversity compared to those with high dietary diversity. This was consistent with research conducted in Gonder, Ethiopia [7], and Kenya [28]. A possible reason could be that a change in dietary diversity could cause a change in nutritional status [41].

In urban kebeles, eating difficulties were significantly associated with malnutrition. Older adults who had an eating problem were 2.8 times more likely to have malnutrition than their counterparts. This study was consistent with the study done in Debre Markos, Ethiopia [10]. The possible explanation is that malnutrition may occur as a result of increasing age and a decrease in the senses of taste, smell, and appetite [25].

Moreover, the presence of chronic illness was a significant factor influencing the nutritional status of older adults living in rural areas. Thus, the risk of malnutrition was 3.8 times higher in older adults with chronic diseases. This finding was consistent with the findings of a study conducted in Eastern Ethiopia [27] and rural Lebanese [32]. This could be because chronic disease can account for nearly half of all malnutrition in older adults [42].

## Conclusions and recommendations

Malnutrition was more prevalent among older adults who lived in rural areas than in urban areas. In the overall study, insomnia, residence, and smoking were significantly associated with malnutrition. DDS, depression, and eating difficulties were significantly associated with malnutrition among older adults living in urban areas, whereas sex, age, depression, chronic disease, and dietary diversity were factors influencing malnutrition in rural areas. Strengthening strategies aimed at addressing nutrition policies, as well as paying attention to the nutritional needs of the older adult population, can help to improve the health and quality of life of older adults. Besides this, public health policies and interventions should address these nutritional risk factors separately in urban and rural areas. 

## Data Availability

For patient confidentiality reasons, the raw data would not be provided. But the summary data is available in the main document.

## Abbreviations

BMI: Body Mass Index
DDS: Dietary Diversity Score
ESPEN: European Society for Clinica, Nutrition and Metabolism
FANTA: Food and Nutrition Technical Assistance
GDS: Geriatric Depression Score
IRB: Institutional Review Board
MNA: Mini Nutritional Assessment
MUAC: Mid Upper Arm Circumference
NGO: Nongovernmental Organization
PAS: Proportional allocation to size
SPSS: Statistical Package for Social Sciences
UN: United Nation
WHO: World Health Organization
